# Effect of S-PRG containing coat on the shear bond strength and the prevention of enamel microcrack formation after orthodontic bracket removal: an invitro study

**DOI:** 10.1186/s12903-026-09062-3

**Published:** 2026-07-04

**Authors:** Mona M Eissa, Mai Akah, Heba Hamza, Elhassan Hassanein

**Affiliations:** 1 Conservative Dentistry Department, Faculty of Dentistry, Cairo University, Cairo, Egypt; 2 Conservative and Restorative Dentistry, Faculty of Dentistry, Galala University, Suez, Egypt; 3 Heinrich Heine Universität Düsseldorf, Düsseldorf, Germany

**Keywords:** PRG BarrierCoat, S-PRG, Shear bond strength, Enamel microcrack, Microcrack prevention, Orthodontic bracket

## Abstract

**Background:**

Among the most common complications after orthodontic treatment are enamel microcracks. These issues arise either from enamel demineralization or during bracket debonding or adhesive remnant removal after bracket removal; thus, the current investigation attempts to assess the impact of S-PRG comprising coat on the shear bond strength of the bonded resin composite and the prevention of enamel microcrack formation following orthodontic bracket removal.

**Methods:**

Fourteen sound, intact human premolars recently extracted were collected. A scanning electron microscope (SEM) was used to assess the enamel surface to exclude teeth with micro-cracks. The teeth were divided randomly into two groups (*n* = 7). In group 1 (control group), the metal bracket was bonded to the etched enamel with (Beauti-Bond xtreme, SHOFU, Japan) adhesive and a thin layer of (Beautifil II Giomer, SHOFU, Japan). In group 2 (intervention group), PRG BarrierCoat^®^ (SHOFU, Japan) was applied on the etched enamel prior to adhesive application. The teeth were then subjected to shear bond strength (SBS) testing using a universal testing machine. SEM and quantitative elemental analysis (weight%) by EDX spectrometry were performed by a calibrated examiner to all teeth to determine the enamel microcrack formation after debonding. The data were analyzed using Fisher’s exact test, independent t-test and the point biserial correlation coefficient. The significance level was set at *p* < 0.05 within all tests.

**Results:**

For SBS, Ca weight, and Ca/P, group (2) had significantly higher mean values than group (1). In contrast, the difference in P weight was not statistically significant. Additionally, the percentage of crack formation in group (1) was higher than that of group (2); however, the difference was not statistically significant. For Ca weight and Ca/P, there was a strong negative correlation with crack formation, with higher mean values significantly associated with the absence of cracks. For P weight, the correlation was not statistically significant.

**Conclusions:**

PRG BarrierCoat^®^ shows promise in enhancing shear bond strength with metal brackets. The high mineral content of PRG BarrierCoat^®^ enhances the integrity of enamel and helps in the prevention of microcrack formation. However, additional long-term clinical trials are required to confirm these findings under dynamic oral conditions.

## Introduction

While orthodontic therapy enhances patients’ oral function and esthetics, it can inadvertently compromise enamel integrity [1]. The development of enamel micro-cracks and fractures remains a major concern during fixed orthodontic therapy. Numerous studies have demonstrated that bracket-debonding often induces enamel micro-cracks, which alters the surface topography, encouraging stain and biofilm accumulation that elevates caries risk and negatively affects esthetics [2].

Heravi et al., in (2008) [3] compared the quantity, length, and orientation of enamel microcracks before and after the metal brackets were removed. They discovered that following debonding, in all groups using three different bracket removal techniques, the quantity and severity of enamel cracks as well as their lengths increased.

Molaasadolah et al., in (2017) [4] found that applying fluoride varnish significantly increased the microhardness of the tooth enamel. Dental materials incorporating Surface Pre-Reacted Glass Ionomer (S-PRG) fillers have recently become accessible for purchase. S-PRG fillers function as bioactive agents that possess the capacity to release fluoride and then recharge it, while also emitting other beneficial ions including strontium, silica, sodium, borate, and aluminum. S-PRG fillers can buffer local acidity, shifting the surrounding medium toward a mildly alkaline state when exposed to acid or water challenges. It was believed that the Na, B, Sr, and F ions liberated from the S-PRG filler were responsible for this impact. The fluoride-releasing coat (PRG Barrier Coat^®^, SHOFU, Japan) incorporating S-PRG fillers, was developed to alleviate dentin hypersensitivity and protect smooth surfaces from caries [5].

Murayama et al. in (2018) [6] demonstrated using optical tomography that PRG Barrier Coat inhibits the demineralisation of bovine enamel. Furthermore [7], studied the effects of three coats (Clearfil SE Protect, PRG Barrier Coat, and Clinpro XT Varnish) on bovine enamel’s nanoindentation hardness and surrounding regions. The coated area was successfully protected against demineralization by all three materials. In 2015, Suzuki et al. [8] investigated the impact of PRG Barrier Coat on primary teeth and demonstrated that it prevented dental caries and inhibited plaque adherence. These findings indicate that PRG Barrier Coat could be beneficial in preventing white-spot lesion development; however, its potential in preventing enamel micro-crack formation has not yet been fully investigated.

An important question to be addressed is how the potentiality of the remineralizing coat would affect the bonding potential of the orthodontic brackets. According to Norevall et al., in (1996) [9] glass ionomer cements which used to benefit from its fluoride release showed less bond strength and a greater rate of fall following orthodontic brackets bonding despite the high fluoride release. So that weighing out the benefits in the crack prevention and substrate quality versus the bondability of the orthodontic bracket should be evaluated.

Among post-treatment complications associated with fixed orthodontic therapy, enamel cracks are commonly reported. Previous studies have indicated that enamel surfaces after debonding may show more pronounced microstructural alterations, including cracking, when compared with untreated teeth [1]. Preservation of enamel integrity during and after bracket removal therefore remains a clinical concern. Thus, if this barrier coat could prevent enamel cracking that occur following orthodontic treatment without jeopardizing the bond strength, this coat will be a beneficial material for orthodontic patients. Therefore, this study’s objective was to assess the impact of S-PRG coat on the shear bond strength of the resin composite bonded brackets and the prevention of enamel microcrack formation after orthodontic bracket removal.

## Materials and methods

The current in vitro investigation was done at the Department of Conservative Dentistry, Cairo University. The protocol of this study was authorized ethically by the Research Ethics Committee, Faculty of Dentistry, Cairo University *(Approval No. 126-7-25)*, and all procedures adhered to institutional and international research guidelines.

### Sample size estimation

Power analysis was used to calculate the sample size using G*Power statistics software (Version 3.1.9.7). A continuous response variable from separate control and experimental subjects -one control or controls for each experimental subject- is used in the investigation. The responses within each subject group were normally distributed with a standard deviation of 1.24 in a prior study by Abuljadayel in (2025) [10]. To be able to reject the null hypothesis that the population means of the experimental and control groups are equal with probability (power) 0.8, we must examine seven experimental subjects and seven control subjects if the true difference between the experimental and control means is 2. This test of the null hypothesis has a Type I error probability of 0.05.

### Preparation of the specimens

Fourteen freshly extracted human premolars indicated for orthodontic extraction were obtained from the Oral Surgery Clinic, Faculty of Dentistry, Cairo University. Teeth exhibiting intact buccal enamel and free from white spot lesions, cracks, fluorosis, restorations, previous orthodontic treatment, or chemical exposure (e.g., hydrogen peroxide) were included. Eligibility was confirmed through review of the patients’ clinical records, and only teeth from patients with no documented history of orthodontic treatment or bleaching procedures were enrolled.

An ultrasonic scaler (Nordent, Germany) was utilized to carefully take out any remaining soft tissue and blood, followed by thorough rinsing with distilled water [11]. Specimens were then kept in 0.1% thymol solution at 24 °C for seven days, and subsequently immersed in normal saline until testing [12].

### Preliminary enamel surface evaluation by SEM

**Fig. 1:**
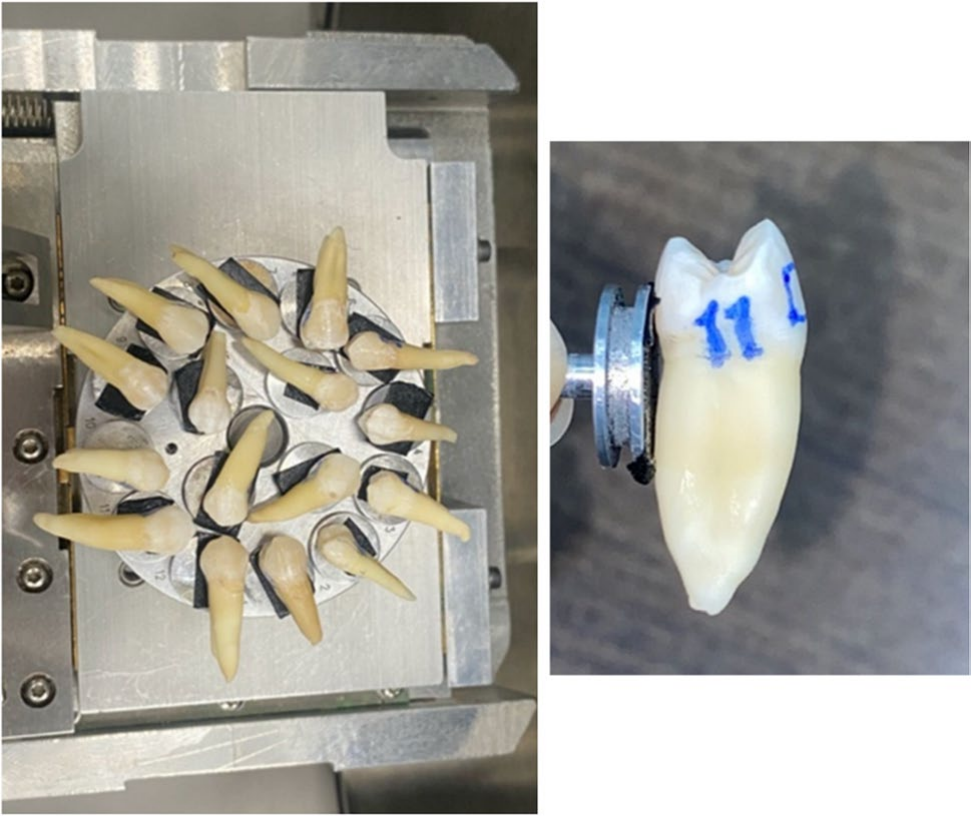
Samples fixed on aluminium stubs with double-stick carbon tape

Preliminary evaluation of the buccal enamel surfaces was conducted with a scanning electron microscopy at Grand Egyptian Museum Conservation Center (SEM, Model FEI Quanta 3D 200i, FEI Company, Netherlands) attached with Energy Dispersive X-ray Unit (EDX, Thermofisher Pathfinder) under operating conditions of accelerating voltage 20 ~ 30 K.V, resolution for Gun.1 nm, using SED (secondary electron detector) and BSED (Back scattered electron detector). SEM examination was performed using low-vacuum operating conditions, which have been utilized to minimize dehydration-related artifacts and crack formation during imaging. Samples were fixed on standard aluminum stubs with carbon adhesive tape and investigated at varying magnifications Fig. 1 [13].

**Fig. 2:**
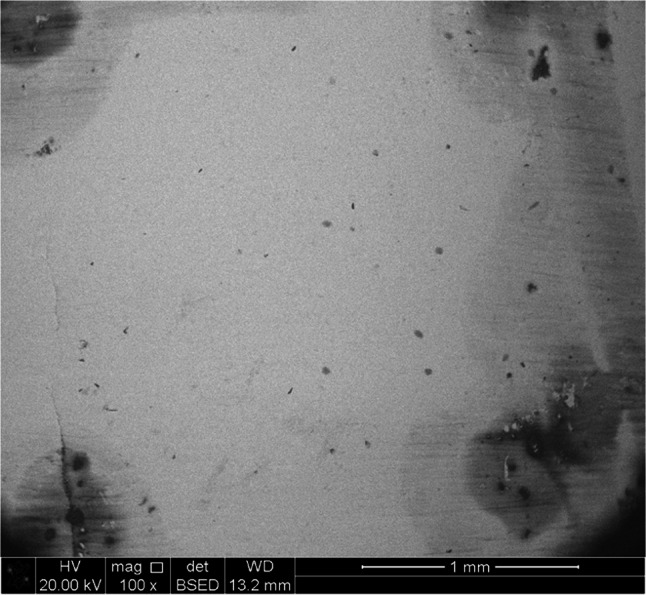
SEM of representative sample of included (crack free) tooth

**Fig. 3:**
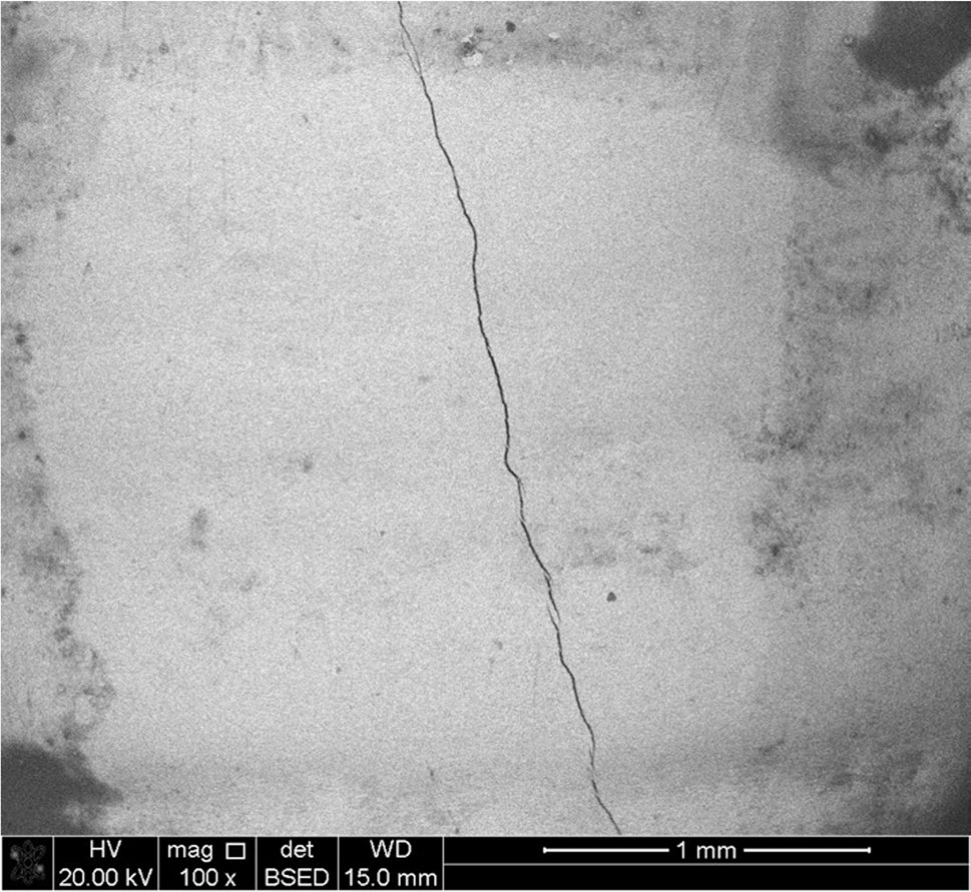
SEM of representative sample of excluded (cracked) tooth

Initial screening for enamel micro-cracks was performed at ×50–×100 magnification. When we were unsure whether a crack was there, we examined the questionable area under a higher magnification to confirm or deny the presence of microcracks. But, teeth exhibiting no visible cracks at these initial magnifications were classified as crack-free and excluded from further high-magnification analysis [2]. Representative included and excluded samples are presented in Figs. 2 and 3.

### Bracket bonding procedure

The premolars were randomly allocated into two groups (*n* = 7) using a computer-generated randomization sequence created with Microsoft Excel (Microsoft Corp., Redmond, WA, USA). Each tooth was assigned a number, and random allocation was performed by an investigator not involved in the experimental procedures to minimize selection bias. The buccal surface of each tooth was properly cleansed before being etched for 30 s using a 35% phosphoric acid gel (Vococid, Voco, Germany). After rinsing the etchant for 20 s, the surface was allowed to air dry for 10 s. A microbrush was used to apply a small layer of adhesive (Beauti-Bond Xtreme, SHOFU, Japan), which was then light-cured for 20 s using an LED curing unit (Elipar FreeLight 2, 3 M/ESPE, USA). A 1-mm layer of Giomer resin (Beautifil II, SHOFU, Japan) was placed on the prepared surface. The chemical compositions of the tested materials are presented in Table 1. The bracket (Vector + MBT, Aditek, Brazil) was positioned following manufacturer instructions using a bracket-holding tweezer. To ensure a highly standardized and reproducible bracket position across all samples, the brackets were positioned precisely at the center of the clinical crown (mid-buccal region), aligning the bracket slot parallel to the long axis of the tooth crown, pressed to ensure firm contact, and excess resin removed. The assembly was then light cured for 10 s to complete bonding [10].

**Table 1:** Materials used in the study

Material	Brand	Composition
Giomer-based Barrier Coat	PRG.Barrier.Coat (Shofu., Kyoto., Japan)	Base: methacrylate monomer, S-PRG filler, glass powder, phosphonic acid monomer, purified water. Activator: TEGDMA, bis-MPEPP, methacrylate acid monomer, catalyzer, carboxylic acid.
Dental universal adhesive agent	Beauti-Bond xtreme (Shofu, Kyoto, Japan)	Bis-GMA, Acetone, carboxylic acid monomer, water, TEGDMA, acid resistant silane coupling agent, organophosphate monomer.
SPRG based restorative material	Beautifil II (Giomer) (Shofu, Kyoto, Japan)	Bis-GMA, aluminum oxide, TEGDMA, silica, pre-reacted glass-ionomer filler, aluminofluoro-borosilicate glass filler, camphoroquinone.

*TEGDMA *Triethyleneglycol dimethacrylate, *bis-MPEPP* Bis-methacryloxy polyethoxypheny propane, *Bis-GMA* Bisphenol A–glycidyl methacrylate

For teeth in the intervention group, although PRG Barrier Coat contains acidic functional monomers and may be used in a self-etch approach, phosphoric acid etching was performed in both groups to standardize the enamel surface treatment protocol and maintain baseline equivalence between the experimental conditions. PRG BarrierCoat^®^ was applied prior to adhesive placement in accordance with the manufacturer’s instructions. After mixing one drop of the activator with the base and applying it uniformly to the etched enamel, it was left undisturbed for a minimum of three seconds and then light-cured for ten seconds. Care was taken to use the mixed material within 2 min of preparation [7]. A single operator handled all bonding procedure. To keep the enamel hydrated until testing, samples were kept in distilled water for 24 h after bonding [10] at room temperature (24°C) to provide a stable and controlled laboratory environment while minimizing additional thermal effects on resin maturation, water diffusion, and possible early hydrolytic degradation [14].

### Shear bond strength testing

**Fig. 4:**
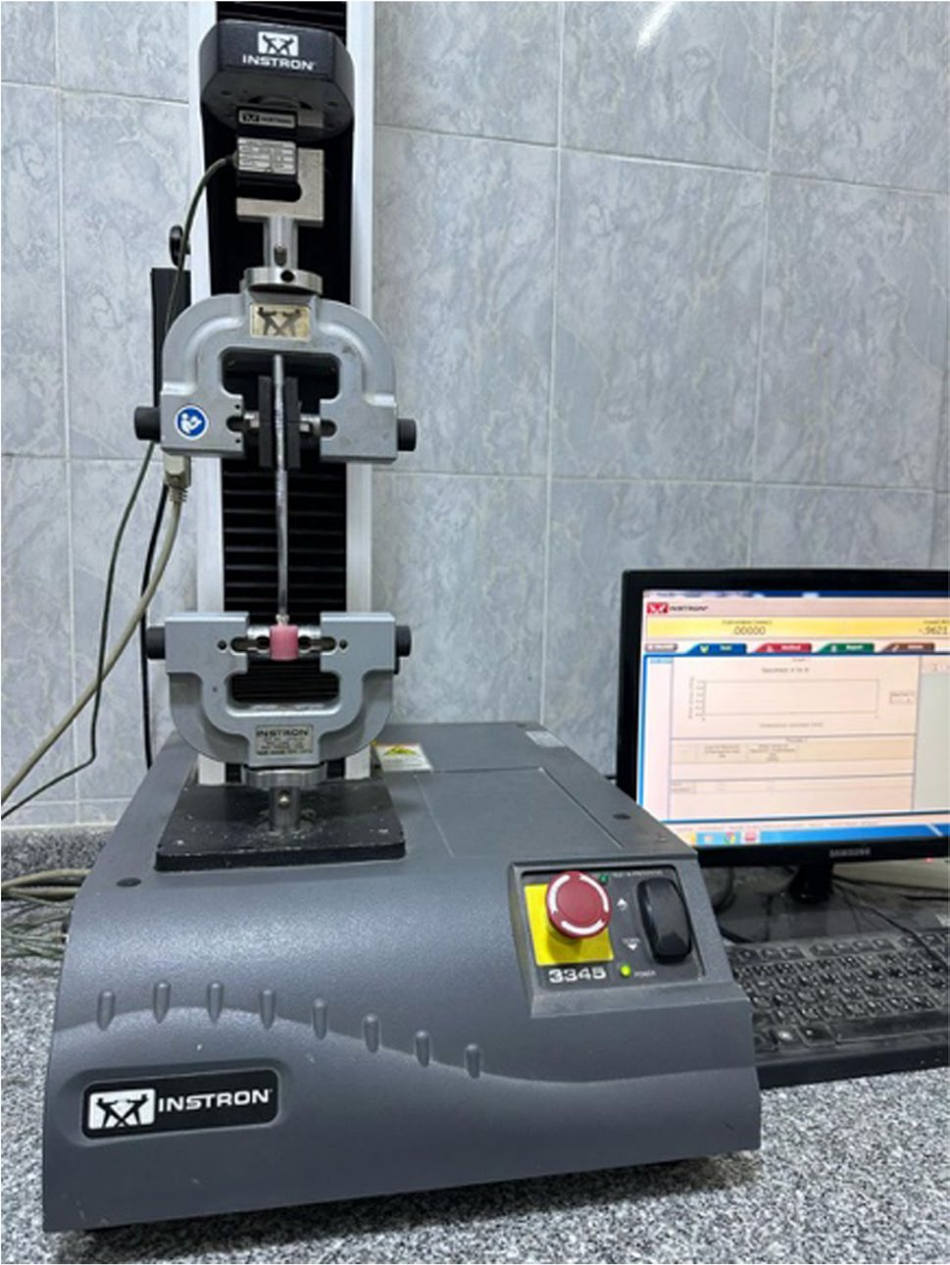
The prepared specimen fixed in the universal testing machine

All samples were inserted in cold-cure acrylic resin (Acrostone, Egypt) so that each crown was adjusted with its buccal surface perpendicular to the block’s base. The bracket’s long axis was aligned with the buccal axial ridge of the tooth to ensure that the applied load would act parallel to the crown’s long axis. The acrylic block containing the specimen was fixed to the lower, stationary head of the universal testing device (Instron 3345, England) Fig. 4.

**Fig. 5:**
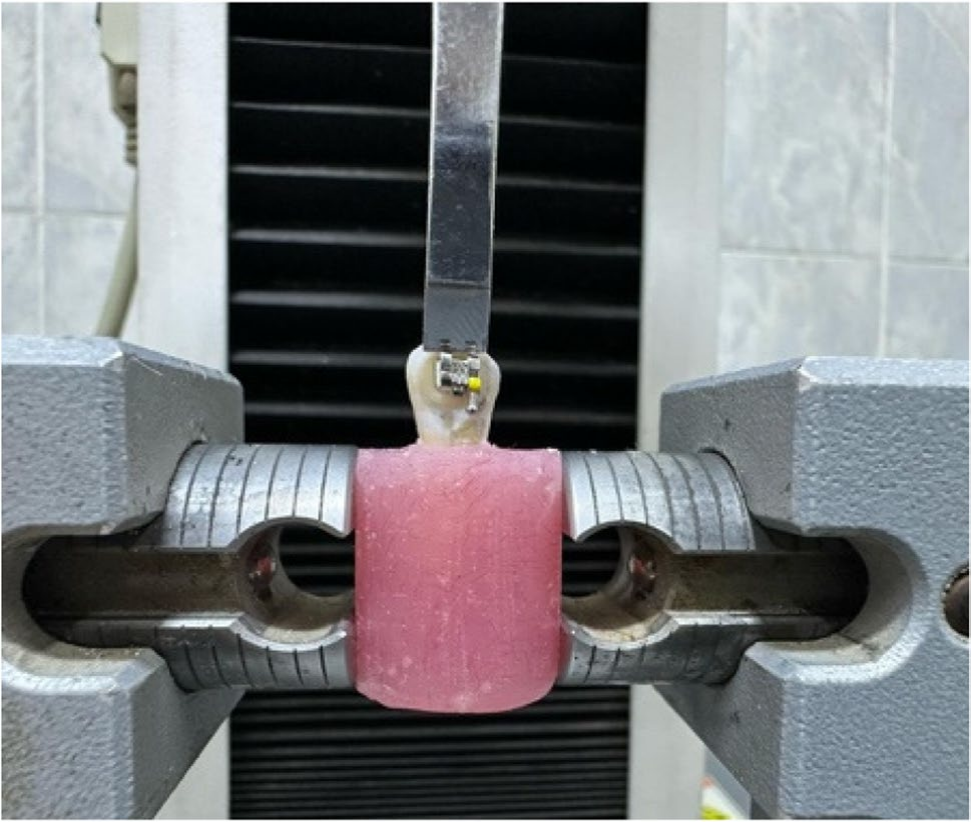
The cheisl blade placed at the bracket/tooth interface

A unibeveled chisel blade with a 0.5-mm width blade was mounted on the upper, movable crosshead of the testing unit. In compression mode, the blade was positioned as close as possible to the interface between the bracket and the tooth surface. A shear load was implemented at a constant crosshead speed of 1 mm/min up to the bracket detached Fig. 5.

The maximum force recorded at the moment of debonding (N) was obtained from the machine output and converted to shear bond strength (MPa) by dividing the force by the bonded area of the bracket base (9.2 mm^2^), using the system software (BlueHill 3, Instron, England) [1].

### Scanning electron microscope after bracket debonding

To avoid causing unnecessary enamel loss during adhesive removal, residual restorative remnant was carefully removed using a One-Gloss polisher (Midi, Shofu, Kyoto, Japan). This system is known to produce a surface morphology comparable to intact enamel, although the process is relatively time-consuming [15]. Then, the buccal enamel surface of every tooth was divided to four equal quadrants to facilitate systematic SEM evaluation of enamel microcracks, as previously described but, images were captured at magnifications ranging from ×100 to ×500, and representative images from different specimens were documented.

All SEM assessments were performed by one calibrated examiner. Imaging was initiated within 30 min of removing the teeth from their storage medium. Although slight dehydration occurred during the vacuum phase of SEM examination, efforts were made to standardize the drying interval for all samples to minimize variability [2].

### Mineral content measurement

**Fig. 6:**
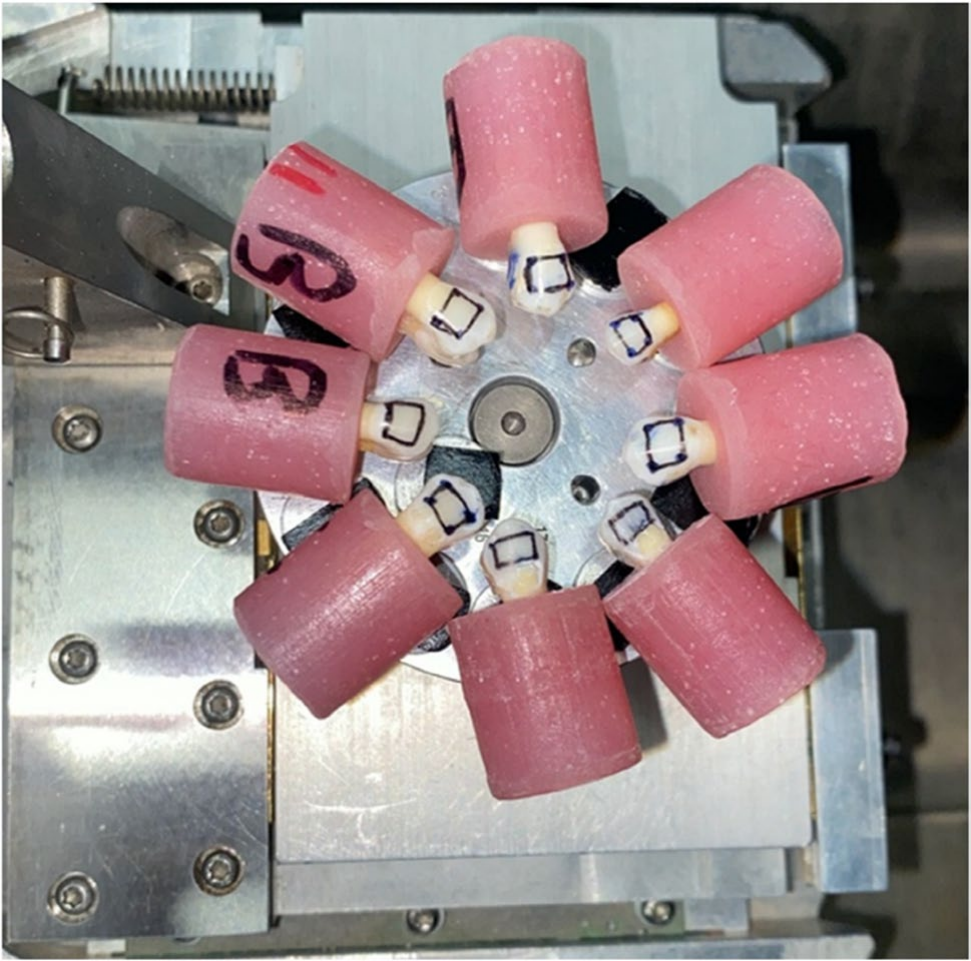
Preparing samples for final SEM/EDX evaluation

Following the SEM examination, the samples were investigated for elemental composition utilizing an energy-dispersive X-ray spectrometer (EDX) (Inspect S, FEI Company, Netherlands). The device was operated at 30 kV accelerating voltage, with a maximum magnification of ×10⁶ and a gun resolution of 1 nm. Each tooth was fixed to a metallic holder using double-sided adhesive tape, ensuring that the prepared enamel window remained oriented upward, as illustrated in Fig. 6. Three separate EDX measurements were taken from the central region of every enamel window, and the mean value was used for statistical analysis. The assessment included quantifying the weight percentages of calcium (Ca) and phosphorus (P) on the enamel surface, followed by calculating the Ca/P ratio in accordance with the protocol described by [16].

### Statistical analysis

Fisher’s exact test was used to examine categorical variables, which were reported as frequency (*n*) and percentage (%). Descriptive statistics for continuous variables comprised the standard deviation (SD) and mean. The distributional characteristics and equality of variances were evaluated by examining the data shape and using the Shapiro-Wilk test for normality and Levene’s test for homogeneity before choosing the right inferential test. An independent t-test was used for comparisons as the continuous results satisfied the requirements of a normal distribution and equal variances among the research groups. The point biserial correlation coefficient was used for correlation analysis. For every analysis, a p-value of less than 0.05 was regarded as indicative of statistical significance. R statistical analysis program, version 4.5.1 for Windows, was used to carry out statistical operations (R Core Team, 2025). R: A statistical computing environment and language. R Foundation for Statistical Computing, Vienna, Austria. URL https://www.R-project.org/.).

## Results

**Fig. 7:**
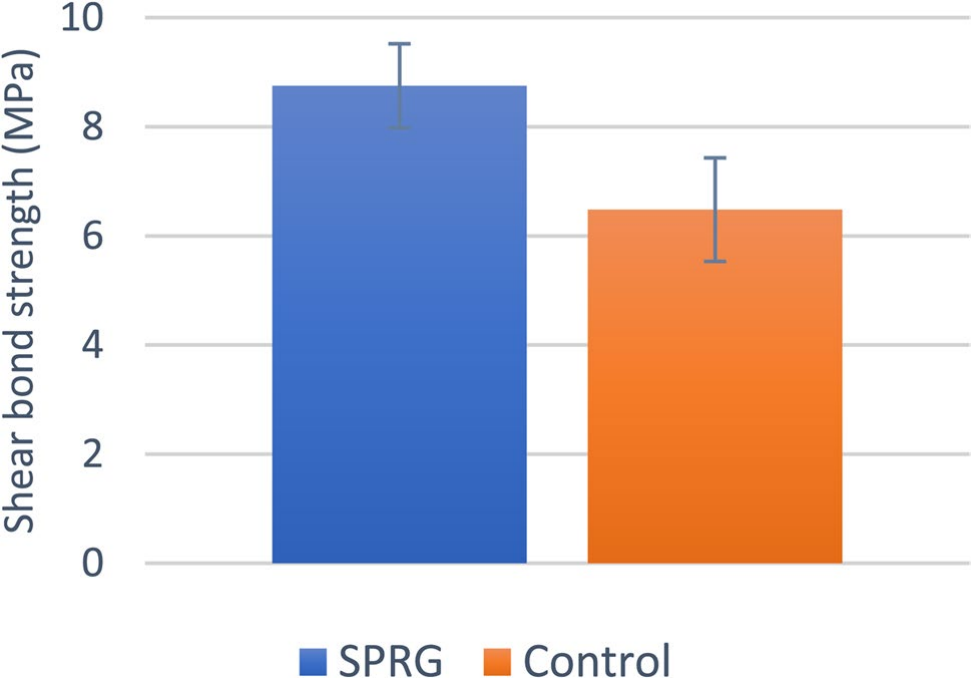
Bar chart showing mean and standard deviation (error bars) for shear bond strength

**Fig. 8:**
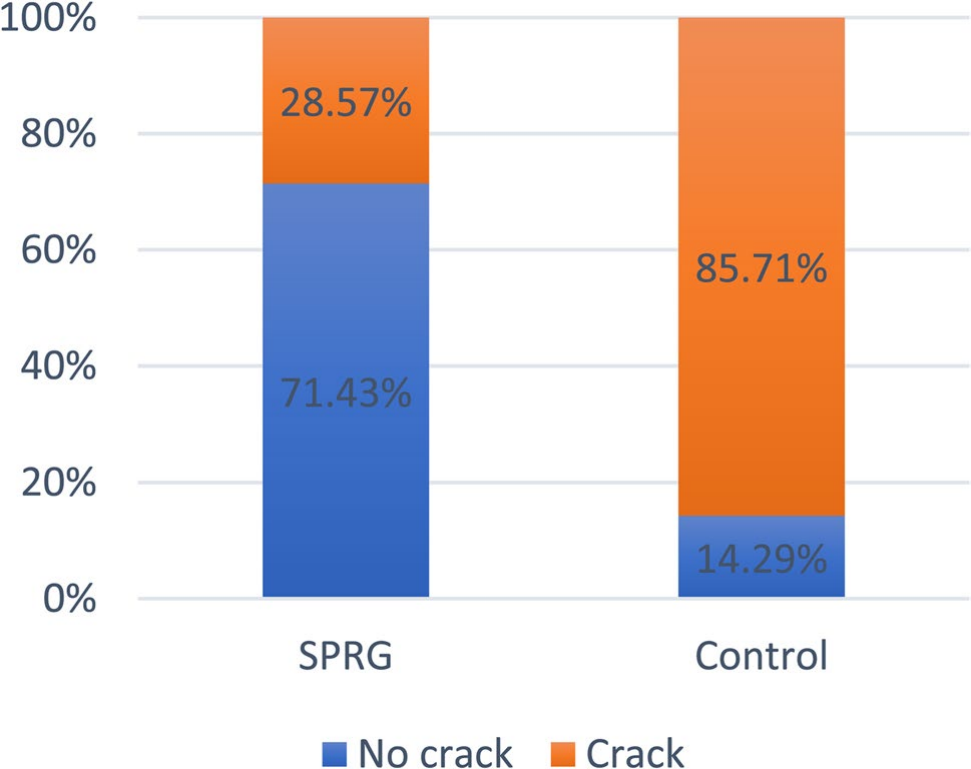
Stacked bar chart showing crack formation distribution

The EDX analysis provided the weight percentages of calcium (Ca) and phosphorus (P) for all specimens, allowing the calculation of the Ca/P ratio for each group. The descriptive statistics and the outcomes of the intergroup comparisons are summarized in Table 2 and displayed in Figs. 7 and 8. Results showed that for shear bond strength, Ca weight, and Ca/P, the intervention group had significantly higher mean values than the control group. In contrast, the difference in *P* weight was not statistically significant.

**Table 2:** Intergroup comparisons

Measurement	Intervention group (SPRG)	Control group	Test statistic	*p*-value
Shear bond strength (MPa) (Mean ± SD)	8.75 ± 0.77	6.48 ± 0.95	4.94	< 0.001*
Ca (weight%) (Mean ± SD)	63.90 ± 3.33	47.33 ± 4.28	8.09	< 0.001*
P (weight%) (Mean ± SD)	35.50 ± 2.98	32.50 ± 5.12	1.34	0.205
Ca/P (Mean ± SD)	1.82 ± 0.24	1.48 ± 0.25	2.56	0.025*
Crack formation [*n* (%)]	2 (28.57%)	6 (85.71%)	NA	0.103

*NA* Not Applicable

^*^Significant (*p* < 0.05)

**Fig. 9:**
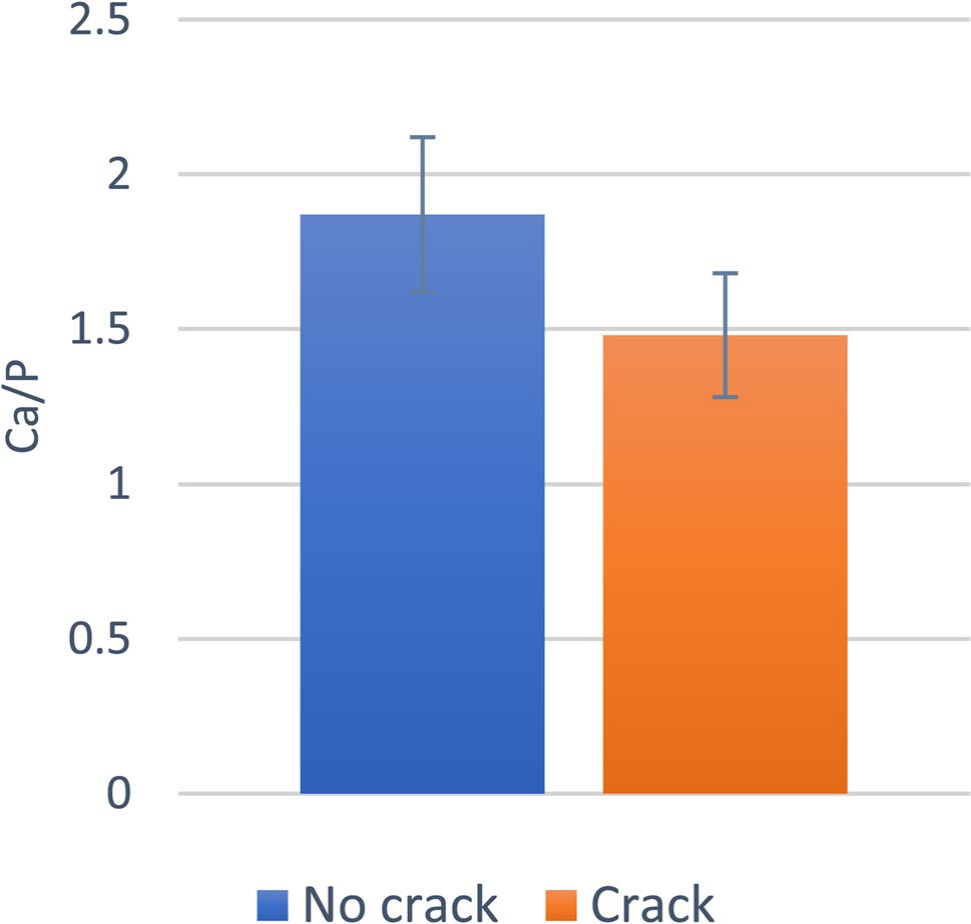
Bar chart showing mean and standard deviation (error bars) for Ca/P in correlation to crack formation

Furthermore, For Ca wight and Ca/P, there was a strong negative correlation with crack formation, with higher mean values significantly associated with the absence of cracks. For P weight, the correlation was not statistically significant. Table 3 and Fig. 9 demonstrate the correlations between crack formation and mineral content.

**Table 3:** Correlations between crack formation and mineral content

Measurement	Mean ± SD		Point biserial correlation	*p*-value
	No crack	Crack		
Ca (weight%)	62.15 ± 6.04	50.71 ± 8.51	-0.63	0.016*
P (weight%)	33.55 ± 4.56	34.33 ± 4.41	0.09	0.751
Ca/P	1.87 ± 0.25	1.48 ± 0.20	-0.69	0.006*

^*^Significant (*p* < 0.05)

**Fig. 10:**
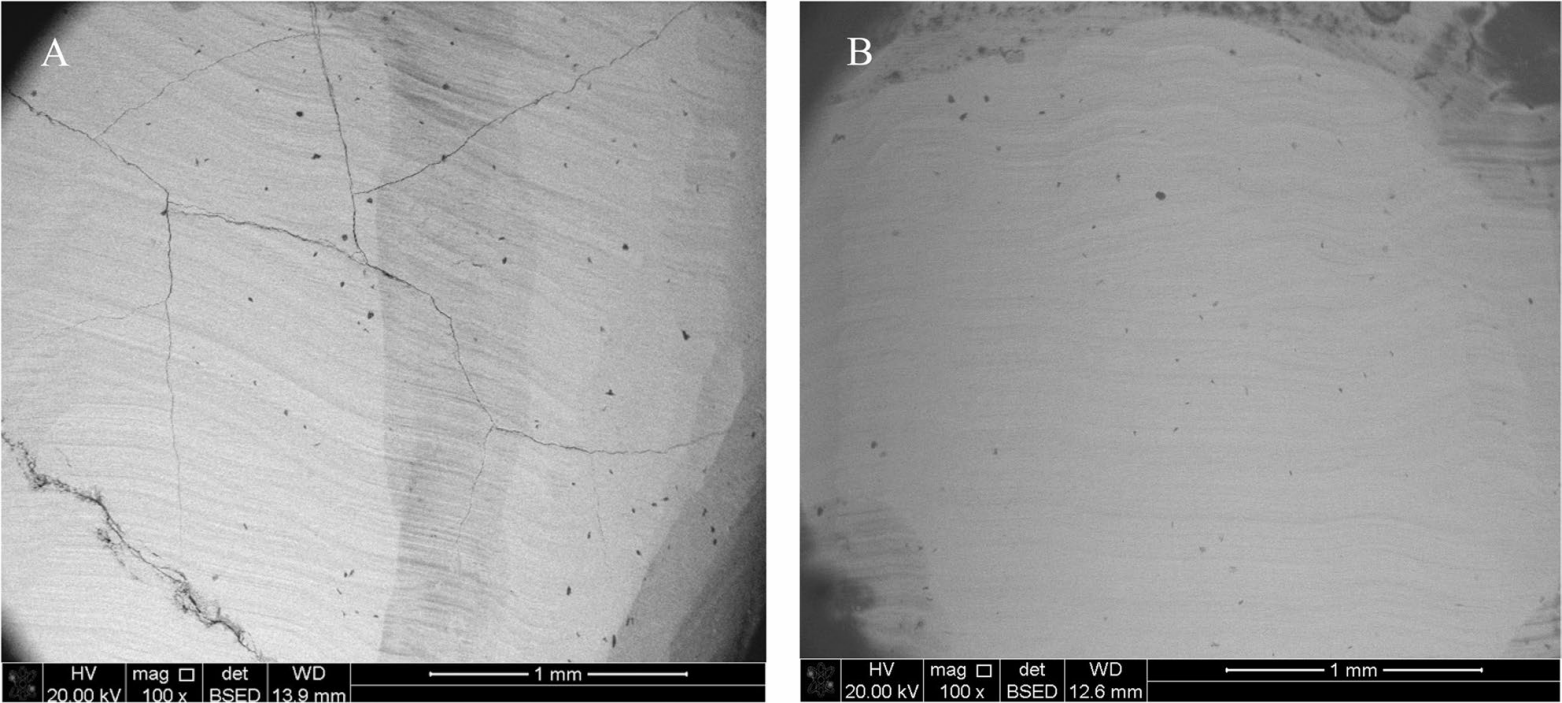
Representative SEM image of low magnification (100X). **A** control group: Shows multiple extensively spreading micro-cracks across the crown, **B** intervention group: Shows a completely clear enamel topography entirely free of visible micro-cracks

**Fig. 11:**
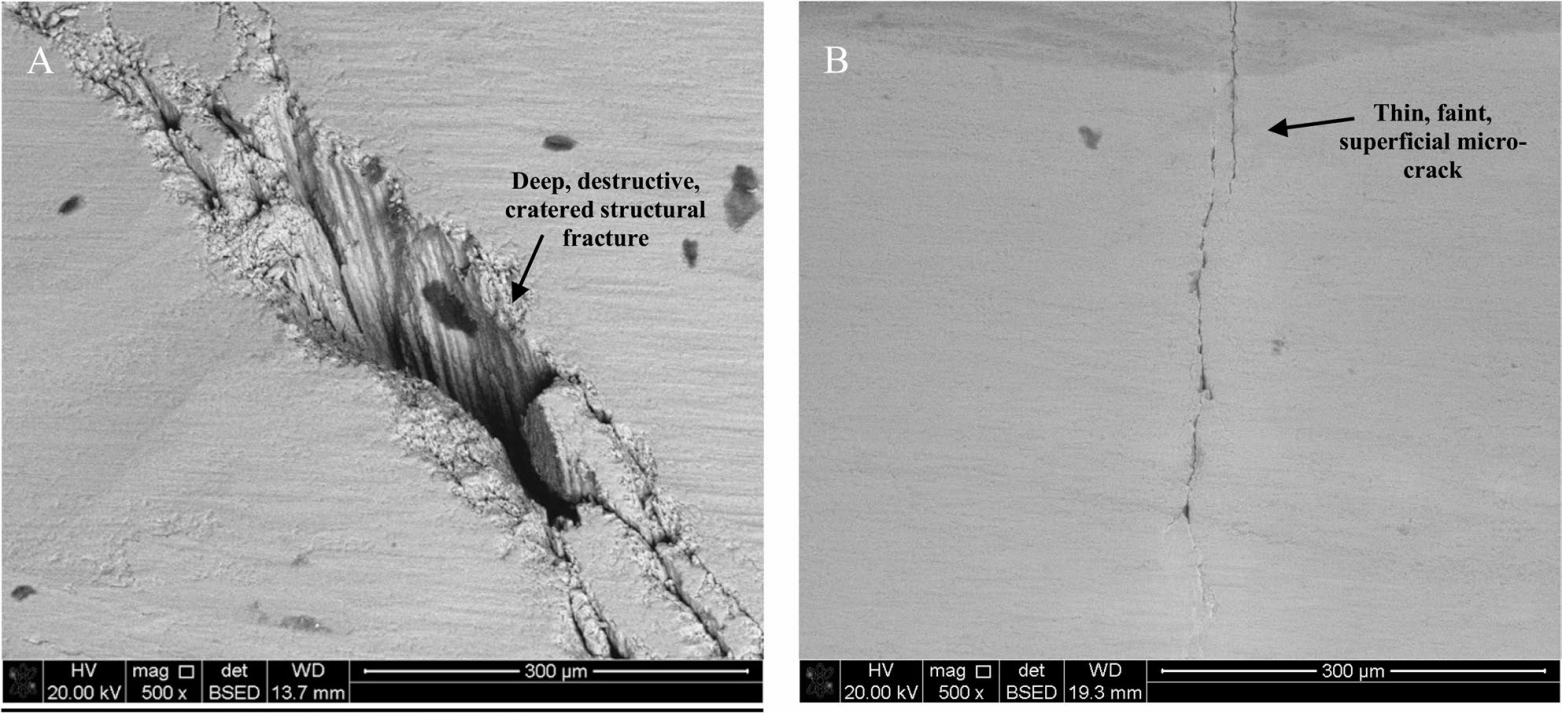
Representative SEM image of high magnification (500X). **A** control group: Shows a deep, destructive, cratered structural fracture with catastrophic collapse, fragmentation, and spalling of the enamel prisms along the margins, **B** intervention group: Shows thin, faint, superficial micro-cracks

Additionally, SEM evaluations revealed distinct numerical and descriptive variations in enamel micro-crack formation post-debonding between the two groups. Within the control group, 85.71% of the sample (6 out of 7 specimens) developed micro-cracks, demonstrating widespread and structurally severe propagation. Specifically, two control specimens exhibited numerous, extensively tracking cracks, while the remaining affected teeth presented with 4 cracks (*n* = 1), 2 cracks (*n* = 1), and 1 crack (*n* = 2). Conversely, the S-PRG intervention group demonstrated a markedly suppressed crack incidence, with only 28.57%of the sample (2 out of 7 specimens) developing micro-cracks. While the total percentage of crack-compromised teeth was numerically lower in the S-PRG group, the difference did not reach statistical significance (*p* > 0.05) Fig. 10. Descriptively, a stark morphological contrast in fracture dynamics was observed between the groups. High-magnification SEM analysis (500X) of the control group revealed severe, deeply cratered structural fractures, where the enamel prisms along the defect boundaries had completely collapsed, fragmented, and spalled away Fig. 11.A. In complete contrast, the two affected specimens in the S-PRG intervention group exhibited micro-cracks that were characteristically faint, thin, and superficial Fig. 11.B.

## Discussion

The primary goal of fixed orthodontic therapy is to establish a bond between the bracket and enamel surface that is strong enough to withstand functional and mechanical stresses throughout treatment yet being easy to remove without damaging the enamel [10]. Shear bond strength (SBS) is affected by multiple variables, particularly the characteristics of the adhesive system and the interactions occurring at various interphases such as between the tooth and composite bonding material [17]. Although many investigations prioritize enhancing the SBS of orthodontic brackets, equal attention must be given to conserving enamel integrity and minimizing permanent damage during debonding procedures [18].

The bioactive dental composites contain components such as fluoride, calcium, and phosphate that enable interactions with biological tissues providing therapeutic benefits that extend beyond simple adhesion [10]. Giomer formulation is based on glass-ionomer technology enhanced by a pre-reacted S-PRG (surface-pre-reacted glass) phase. Upon exposure to polyacrylic acid, fluoraminosilicate glass particles react chemically and integrate into the resin matrix, enabling sustained fluoride release [19].

According to Pont et al. in (2010) [20], some degree of enamel damage following bracket removal is unavoidable. A more recent investigation found that new enamel micro-cracks developed in 6 of 15 examined teeth (40%) after debonding [2]. Building upon this concept, Peng et al. in (2022) [1] adopted the idea of applying enamel coatings utilizing self-assembling antimicrobial peptides to prevent demineralization associated with orthodontic appliances. Their findings demonstrated that such coatings represent a promising strategy for limiting the occurrence of white spot lesions and enamel micro-cracks. Inspired by this approach, the present study explored the use of a mineral-based coating to protect enamel during orthodontic treatment.

Our study assessed the enamel surface after 24 h to capture the early-stage interaction between the material and enamel substrate. This time point is clinically relevant, as ion release and surface interactions from surface pre-reacted glass (S-PRG) fillers are known to occur immediately after application, with measurable physicochemical effects reported within short-term exposure periods. Importantly, previous in vitro studies have demonstrated that enamel microhardness changes can already be detected within the first 24 h of exposure to ion-releasing materials [21]. Therefore, the 24-hour evaluation in the present study was selected to reflect the early biological response phase rather than long-term material maturation.

Every group in the current investigation showed bond strengths that were within the range that was considered clinically acceptable. The most commonly reported bond strengths for orthodontic brackets are between 6 and 8 MPa [22]. While excessively high bond strength increases the danger of enamel injury during debonding, lower bond strength can lead to repeated bond failures [23]. The bond strength should remain below the enamel’s maximum tensile strength limit for safe bracket removal without enamel cracking or fractures. According to reports, enamel’s tensile strength ranges from 11 to 25 MPa [24].

A key finding of this study was the significantly higher Shear Bond Strength demonstrated by the intervention group, which utilized an alternative protocol involving the application of an S-PRG coat over a pre-etched enamel surface. As outlined in our methodology, phosphoric acid etching was applied uniformly to both groups to standardize the enamel surface treatment protocol and eliminate baseline substrate variability. While the S-PRG Barrier Coat contains acidic functional monomers capable of a self-etch approach, relying on this capability exclusively in the intervention group would have introduced methodological bias. Because the enamel conditioning was identical across both groups, the superior bond performance of the intervention group might be attributed to the specific synergy between the pre-etched enamel and the subsequent S-PRG coating. Acid etching creates high-energy micro-porosities, optimizing the enamel for mechanical interlocking [14]. When the fluid resin of the S-PRG coat is applied over this highly receptive surface, it can deeply infiltrate the open prism patterns. Furthermore, S-PRG fillers are highly ionic and form a stable network at the adhesive interface [13].

Consequently, while this protocol introduces an extra clinical step contrary to the manufacturer’s simplified instructions, combining enamel etching with an S-PRG coating appears to be highly effective in maximizing orthodontic bond strength. This outcome aligns with findings from Spinola et al. in (2020) [25], who observed that the PRG Barrier Coat achieves strong adhesion through combined mechanical and chemical bonding. This giomer-based coating contains S-PRG filler particles within a resin matrix, enabling fluoride and other ions release that supports remineralization and enamel protection. Conversely, Abuljadayel in (2025) [10] reported that although several bioactive adhesives yielded satisfactory bond strengths at the enamel–bracket interface, the Beautifil II material demonstrated comparatively lower values.

Enamel is composed of approximately 95 wt% mineral, primarily an impure form of calcium hydroxyapatite. These crystals, with specific hierarchical microstructure is designed to withstand abrasive and mechanical forces. Enamel becomes more susceptible to deformation and crack propagation when mineral content decreases [26]. Enamel hardness is also strongly correlated with chemical composition. Regions with elevated calcium and phosphorus concentrations exhibit greater nanohardness, whereas increased sodium and magnesium levels are associated with reduced hardness [27]. Reduced hardness has likewise been reported in areas with lower Ca and P content [28].

In the current study, the control group exhibited a higher incidence of enamel cracking compared to the intervention group. In the control group, the standard bonding protocol involves phosphoric acid etching, which creates a demineralized, porous, and high-energy surface. While necessary for resin micromechanical interlocking, this process temporarily lowers the enamel’s surface stiffness and elastic modulus. When subjected to the high-torque stresses of bracket debonding, this “brittle” etched layer is highly susceptible to the initiation and propagation of micro-cracks [29].

While the significant reduction in crack formation observed in the intervention group may be linked to the timing of the coating application within the present methodology. By applying the S-PRG-based mineral coating immediately after acid etching but prior to bracket bonding, the enamel was protected at its most vulnerable state. By the time debonding occurs (even at the 24-hour), these structural voids are already occupied by a reinforced resin matrix. Also, adhesives containing S-PRG fillers have been shown to release fluoride and additional ions capable of penetrating adjacent enamel and dentin, producing an acid-base resistant zone at the bonded interface [30, 31]. In the present study, the coating likely functioned as a bioactive infiltrant that penetrated the etched enamel prisms and polymerized to form a reinforced hybrid layer. This layer may have sealed the etched surface and physically reinforcing the tissue’s hierarchical microstructure and distributed mechanical stresses more uniformly across the enamel–adhesive interface, thereby reducing crack propagation.

Beyond the clear numerical disparities, distinct descriptive and morphological variations in enamel microcrack formation were observed between the two groups post-debonding. While the control group displayed distinct, sharply defined structural fractures, the cracks in the S-PRG pretreated enamel were highly attenuated and superficial. This distinct variation in crack morphology and frequency suggests that the increased calcium and phosphate content induced by the S-PRG coating successfully elevated the fracture toughness and cohesive strength of the enamel matrix, effectively dampening mechanical stress propagation during bracket removal.

Our chemical analysis further supports this interpretation. A strong negative correlation was observed between calcium (Ca) weight, Ca/P ratio, and crack formation, with higher values significantly associated with the absence of cracks. This finding is consistent with Alsayed et al. (2016) [7], who demonstrated that S-PRG coating materials improved enamel resistance to demineralization through both physical and chemical protective mechanisms. They also reported enhanced hardness in adjacent uncoated enamel areas, likely due to diffusion of ions released from the S-PRG filler, including fluoride, strontium, boron, sodium, silicon, and aluminum. Silicon and aluminum contribute to the structural glass network, whereas fluoride and strontium act as modifying ions that enhance enamel resistance [7]. Also, Kawamura et al. in (2017) [29] observed that enamel treated with PRG Barrier Coat or Clinpro XT varnish exhibited higher hardness and elastic modulus values within 1–11 μm of the surface and within 300 μm of the coating margin. These findings suggest that ion release from coating materials may inhibit demineralization and preserve enamel mechanical properties.

The higher Ca/P ratio in the intervention group does not necessarily represent the growth of “new” crystals. Instead, it indicates that the S-PRG coating successfully protected the native mineral content from being further compromised during the debonding phases. Also, S-PRG technology is characterized by a “burst effect” of ion release (Fluoride, Strontium, Boron) upon contact with moisture. Within the first 24 h, these ions associate with the etched enamel surface to form an Acid-Base Resistant Zone [10, 30]. While this may not be a change in the bulk crystalline structure, it represents a significant chemical enrichment of the surface layer, which explains the increased resistance to damage observed in our study.

Interestingly, while calcium levels and Ca/P ratios were strongly associated with enamel integrity in the present study, phosphorus content alone showed no statistically significant correlation with crack formation. This observation agrees with Alkattan et al. (2018) [32], who suggested that surface calcium concentration and Ca/P ratio are more sensitive indicators of enamel resistance to demineralization and structural breakdown than phosphorus levels alone.

### Limitations and future directions

Finally, it is important to recognize that this study was performed in-vitro. Such circumstances do not accurately capture the complicated nature of the oral environment, including variations in saliva, temperature fluctuations, and functional forces. Therefore, bracket debonding behavior in vivo may differ from laboratory outcomes. While the 24-hour results demonstrate a clear protective effect of the mineral-based coating against debonding trauma, the long-term sustainability of this reinforced interface remains to be seen. Future research should incorporate clinical trials, thermocycling, and long-term evaluation to validate these findings under real patient conditions. Furthermore, future studies may benefit from using confocal laser scanning microscopy (CLSM) as a more suitable non-vacuum technique for enamel crack assessment, in addition incorporating failure mode analysis may provide more comprehensive evaluation.

## Conclusion

Within the limitations of this in vitro study, the application of a PRG barrier coat before orthodontic bracket bonding appeared to contribute to enamel surface preservation without compromising the effectiveness of the bonding procedure. These findings suggest that the use of a PRG barrier coat may represent a promising adjunctive strategy for protecting enamel during orthodontic treatment. Nevertheless, the clinical significance of these observations remains to be established, and further well-designed in vivo studies with long-term follow-up are required to confirm their clinical applicability.

## Data Availability

Upon request, the corresponding author will provide all datasets created and examined during the current work.

## Abbreviations

S-PRG: Surface Pre-Reacted Glass Ionomer
SBS: Shear Bond Strength
SEM: Scanning electron microscope
EDX: Energy Dispersive X-ray
BSED: Back Scattered Electron Detector
Ca: calcium
P: phosphorus
SD: Standard Deviation
